# Advancements in biotransformation pathway prediction: enhancements, datasets, and novel functionalities in enviPath

**DOI:** 10.1186/s13321-024-00881-6

**Published:** 2024-08-06

**Authors:** Jasmin Hafner, Tim Lorsbach, Sebastian Schmidt, Liam Brydon, Katharina Dost, Kunyang Zhang, Kathrin Fenner, Jörg Wicker

**Affiliations:** 1 University of Zürich, Zürich, Switzerland; 2 Eawag, Dübendorf, Switzerland; 3 enviPath, Mainz, Germany; 4 Bayer AG, Crop Science Division, Mohnheim, Germany 40789; 5 School of Computer Science, University of Auckland, Auckland, New Zealand

**Keywords:** Metabolic pathways, Machine learning, Biodegradation pathway prediction, Biodegradation database

## Abstract

**Abstract:**

enviPath is a widely used database and prediction system for microbial biotransformation pathways of primarily xenobiotic compounds. Data and prediction system are freely available both via a web interface and a public REST API. Since its initial release in 2016, we extended the data available in enviPath and improved the performance of the prediction system and usability of the overall system. We now provide three diverse data sets, covering microbial biotransformation in different environments and under different experimental conditions. This also enabled developing a pathway prediction model that is applicable to a more diverse set of chemicals. In the prediction engine, we implemented a new evaluation tailored towards pathway prediction, which returns a more honest and holistic view on the performance. We also implemented a novel applicability domain algorithm, which allows the user to estimate how well the model will perform on their data. Finally, we improved the implementation to speed up the overall system and provide new functionality via a plugin system.

**Scientific contribution:**

The main scientific contributions are the development of a pathway prediction model applicable to diverse chemicals, a specialized evaluation method for holistic performance assessment, and a novel applicability domain algorithm for user-specific performance estimation. The introduction of two new data sets, and the creation of links to EC classes make enviPath a unique resource in microbial biotransformation research.

## Introduction

**Fig. 1:**
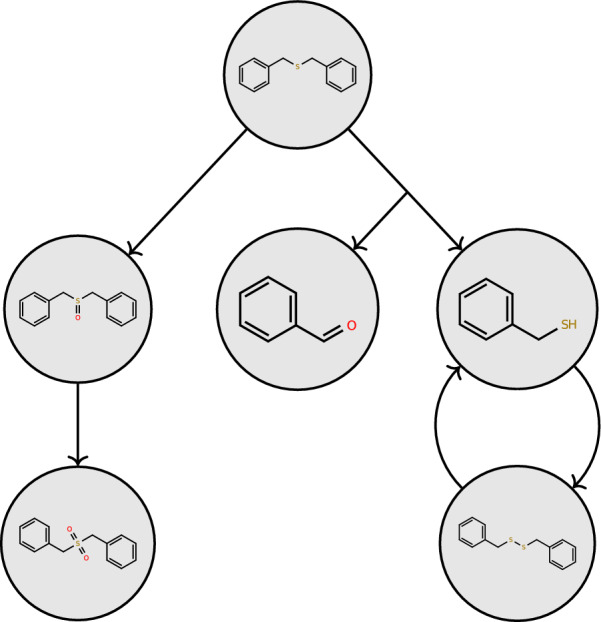
The pathway *Benzyl Sulfide* from the *Eawag-BBD* package

enviPath is a unique resource that focuses on microbial biotransformation pathways of primarily xenobiotic chemical compounds [1]. Since its initial release in 2016, enviPath has become widely adopted in research and industry. enviPath distinguishes itself from other metabolic pathway databases (e.g., KEGG [2–4]) by focusing on chemicals that are man-made xenobiotics and are known or suspected environmental contaminants. The primary objective of enviPath is to offer details on experimentally observed enzyme-catalyzed reactions of environmental contaminants, which can be useful for several applications such as bioremediation, chemical risk assessment, and analysis of contaminants and their transformation products in the environment. Figure 1 shows the *Benzyl Sulfide* pathway from the *Eawag-BBD* data package [1, 5] as an example.

**Fig. 2:**
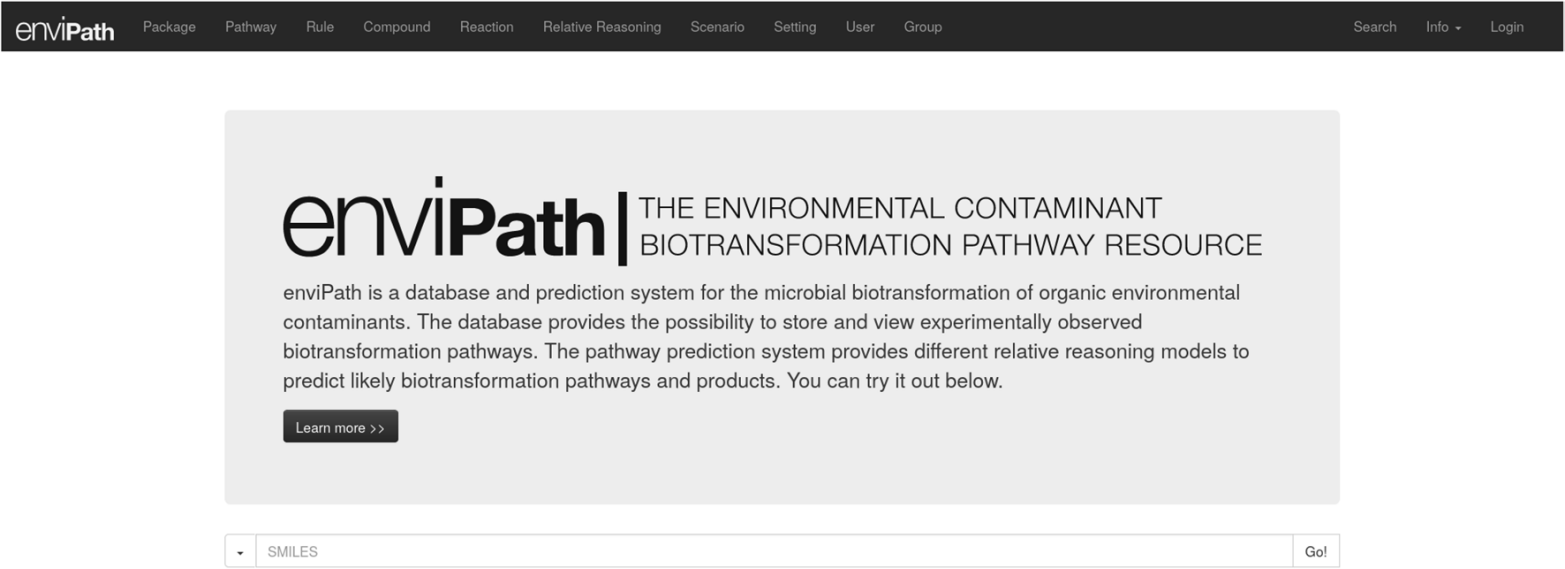
The enviPath home page

enviPath also provides a pathway prediction engine [6–8] to predict microbial biotransformation pathways. The system uses biotransformation rules to detect functional groups in organic compounds, and prioritization rules to fine-tune the predictions of corresponding reactions and products. Both types of rules are based on reactions found in the enviPath databases. Figure 2 shows the home page of enviPath with the prompt that can be used to submit compounds to the prediction engine.

Over the past years, we have made continuous improvements to enviPath, in terms of the data sets, the algorithm, and capabilities of the prediction engine. In particular, we have introduced two new data sets, namely *Eawag-Soil* [9] and *Eawag-Sludge* [10], in addition to our primary data set, *Eawag-BBD*. *Eawag-Soil* provides pathway information from soil degradation studies, extracted from pesticide registration dossiers (draft assessment reports, DAR) that have been made publicly available by the European Food Safety Authority (EFSA). It also includes details about different experimental conditions and, when available, a biotransformation half-life (DT$_{50}$) value. The *Eawag-Sludge* package contains pathways and kinetic information regarding microbially mediated transformation processes in biological wastewater treatment, along with details about experimental conditions and supplementary information such as the source of the sludge used in the biotrasformation experiment. The information has been extracted from various scientific publications across different journals.

To aid in understanding which enzymes can facilitate certain biotransformation reactions in environmental microbial communities, we established connections between transformation rules and EC classes. Most existing tools developed to predict enzymes that may catalyze a given transformation reaction have been trained on natural metabolic reactions [11–13]. Hence, they are not very effective for predicting enzymes involved in contaminant biotransformation. To address this, we introduced a new feature called enviLink [14], which establishes connections between generalized biotransformation rules and 3$^{rd}$-level EC classes sourced from the *Eawag-BBD* data and KEGG.^1^

We made several improvements to the prediction engine. Most importantly, we implemented a more comprehensive evaluation approach for pathway predictions that considers entire pathways rather than isolated reactions [15], we provide an applicability domain for biodegradation predictions [16], and we enhanced the computational efficiency of prediction and model training.

## Construction and content

**Fig. 3:**
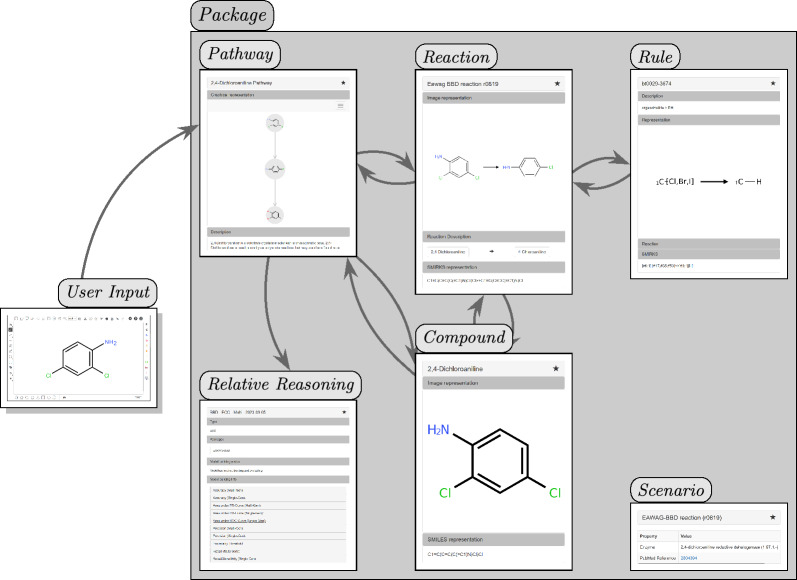
Schematic representation of the enviPath framework illustrating entity interactions. User input is provided through a visual editor or via SMILES notation within the web interface. The computational *Model* predicts pathways based on this input. The depicted pathway is an illustrative example sourced from the database and not an actual predictive output. The pathway consists of *Reactions* (represented along edges) and *Compounds* (situated at nodes). A *Reaction* is associated with a corresponding *Rule*, which generalizes across multiple similar *Reactions*. In cases where *Reactions* are manually introduced, *Rule*s can be added to describe the general biotransformation mechanism. Furthermore, all entities incorporate supplementary information referred to as *Scenario*. Each entity is further organized within a *Package* for systematic organization and accessibility

enviPath includes various entities and relationships. Figure 3 visualizes the core database schema. Users can input data using a web form or SMILES input, and the Prediction Engine will predict a pathway, or a stored pathway from the database will be shown if available. The predicted or stored pathway is connected to Reactions and Compounds, with Reactions potentially linked to the Rule used to predict them. When Reactions are manually inserted, links to Rules can be established to indicate which Reaction the Rule was generalized from. Each entity in the system has additional information called Scenario, which can include details like the corresponding PubChem [17] entry, the enzyme involved in the reaction, or a set of experimental and environmental conditions. All entities are organized into Packages.

### Data

We continuously update and add data packages containing information on microbial contaminant biotransformation pathways and kinetics, including appropriate meta-data describing the specific study conditions as *Scenarios*. Currently, we host the most up-to-date, well curated and annotated sets of microbial biotransformation pathways and half-life data for contaminants in soil and activated sludge. For an overview of the numbers of entities, see Table 1. While *Eawag-Soil* has been introduced and described extensively in [9] and [1], below more details are given for the recently published *Eawag-Sludge* package.

**Table 1:** Data statistics of the main packages in enviPath

	BBD [1]	Soil [9]	Sludge [10]
Compounds	1399	1780	1070
Compounds with half-lives	–	895	172
Reactions	1480	2447	521
Pathways	219	317	184

We give the number of *Compounds*, *Reactions*, and *Pathways* per dataset. Additionally, we give the number or compounds that have a half-life associated with them. Since the initial enviPath release with only the BBD package, we strongly increased the data set size and provide a far more diverse set of pathways and reactions

#### Eawag-Soil

During a recent analysis and curation of *Eawag-Soil* data [9], several errors were detected and fixed. A continuously updated record of changes is kept at our Wiki.^2^

#### Eawag-Sludge

The *Eawag-Sludge* package is a compilation of biodegradation studies in activated sludge comprising results from 27 scientific articles, published between 1999 and 2023, see Table 1 for statistics. The reporting options for activated sludge-specific experimental metadata consist of acidity (pH), addition of nutrients, biological treatment technology, bioreactor type and volume, spike compound concentration and solvent, inoculum source, location and purpose of the wastewater treatment plant, nitrogen content, redox conditions, sludge retention time, total suspended solids (TSS), and type of aeration. In terms of chemical space, *Eawag-Sludge* provides biodegradation pathways and kinetcis for a diverse set of chemicals including pharmaceuticals, pesticides, and industrial chemicals.

## Utility and discussion

Since the first implementation of the prediction engine [7], enviPath has undergone multiple iterations that improved the prediction of pathways.

### Updates on the prediction engine

Important steps in improving the prediction engine were the improvement due to learning dependencies among the transformation rules [8] and extending the source data [9]. Further, we implemented a more realistic, holistic evaluation [15] of pathway prediction and tailored an applicability domain specifically for metabolic pathway prediction [16]. In the following, we will focus on the improvements that we implemented since the initial release and publication of enviPath [1].

#### Multi-generation evaluation

Biotransformation prediction engines predict pathways by iteratively applying transformation rules to a compound of interest. The easiest way to evaluate the performance of a prediction algorithm is by assessing its ability to reconstruct known reactions without predicting too many products that are not experimentally observed. However, such a single-generation evaluation has only a limited capacity of evaluating the ability to predict whole pathways. Multi-generation evaluation is crucial for accurately assessing environmental biotransformation models, as it captures the complexity of pathways and transient intermediates often overlooked in single-generation evaluations, preventing misleading assessments and incorrect labeling of transformation products as false positives. The issue of single-generation evaluation is of particular importance for environmental biotransformation pathways, as such pathways are often not reporting short-lived, transient intermediates that are difficult to detect analytically. As a consequence, processes that take several reaction steps are sometimes reported as one multi-step reaction. Not accounting for these intermediates in the pathway evaluation leads to misleading assessment of model performance.

**Fig. 4:**
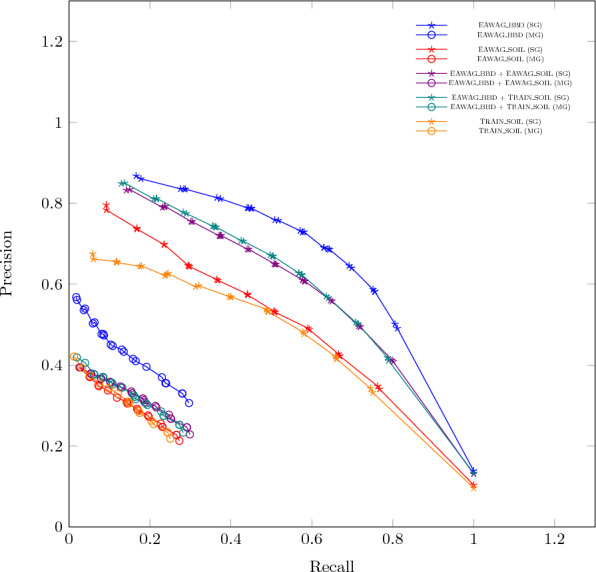
Precision-Recall curves for different holdout experiments. Each of the experiments keeps A random subset of the data as test set, repeating the experiments 100 times. Then both single-generation and multi-generation evaluation is performed. The legend gives the data sets used in the evaluation. Details on the specific composition of the data sets are given in our previous publication [15]

Another important issue addressed by the proposed evaluation framework are higher-generation transformation products. In branched pathways, concentrations of higher-generation transformation products are expected to be significantly lower than any initially spiked parent compound concentrations, up to a point where transformation products might not be detectable anymore. This may lead to an incorrect evaluation as false positives even if the model correctly predicts the occurrence of this transformation.

**Fig. 5:**
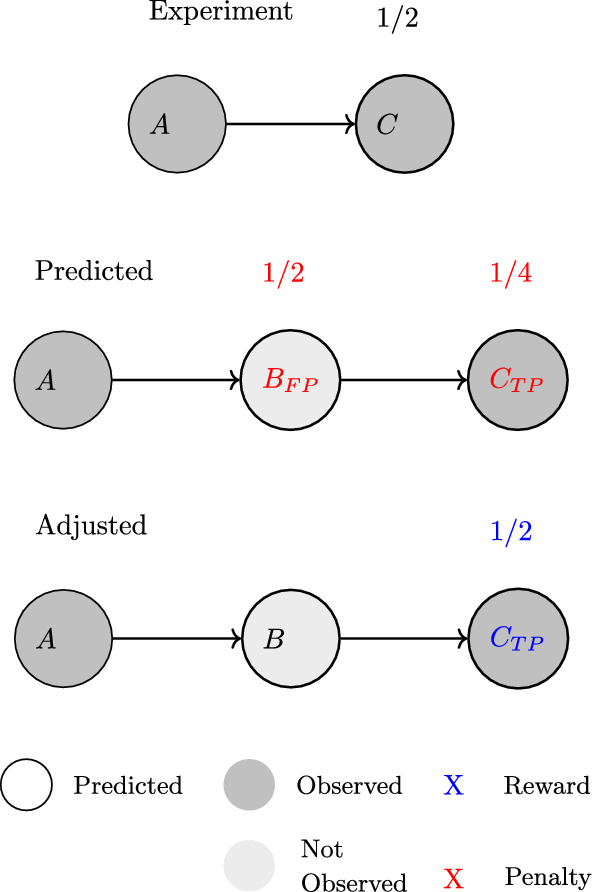
The depth adjustment process according to intermediate metabolites determined in the predicted pathway. Compounds *A* and *C* are present in both observed and predicted pathways, which allows compound *B* to be identified as an intermediate metabolite. It can be ignored and the depth-associated weight for scoring can be adjusted accordingly for compound *C*. Figure taken from our previous work [15]

To address the issue of evaluating whether predicted pathways are coherent with experimental observations, a new multi-generation evaluation framework has been implemented in enviPath [15]. It includes a scoring system that puts decreasing weight on higher generation transformation products and does not penalize the prediction of intermediates (see Fig. 5).

The finite number of transformation rules that do not cover the full transformation space of the reference data leads to another challenge. The single-generation performance of models trained on different packages (i.e., different pathways, reactions, compounds, or rules) are not comparable. The models are based on a specific set of rules and reference transformations. Hence, properties of the prediction task, such as class distributions or number of targets, depend on the training package. The new multi-generation approach overcomes this problem by being model agnostic, that is it allows the comparison of models trained on different packages. This allows for example to study the effect of adding additional transformation rules.

Figure 4 gives an example evaluation result for both single-generation and multi-generation measures. The figure shows that multi-generation evaluation returns lower precision and recall values compared to single-generation evaluation. This highlights the importance and impact of effects such as the loss of complete substructures in the pathways when specific reactions are not predicted.

#### Applicability domain

**Fig. 6:**
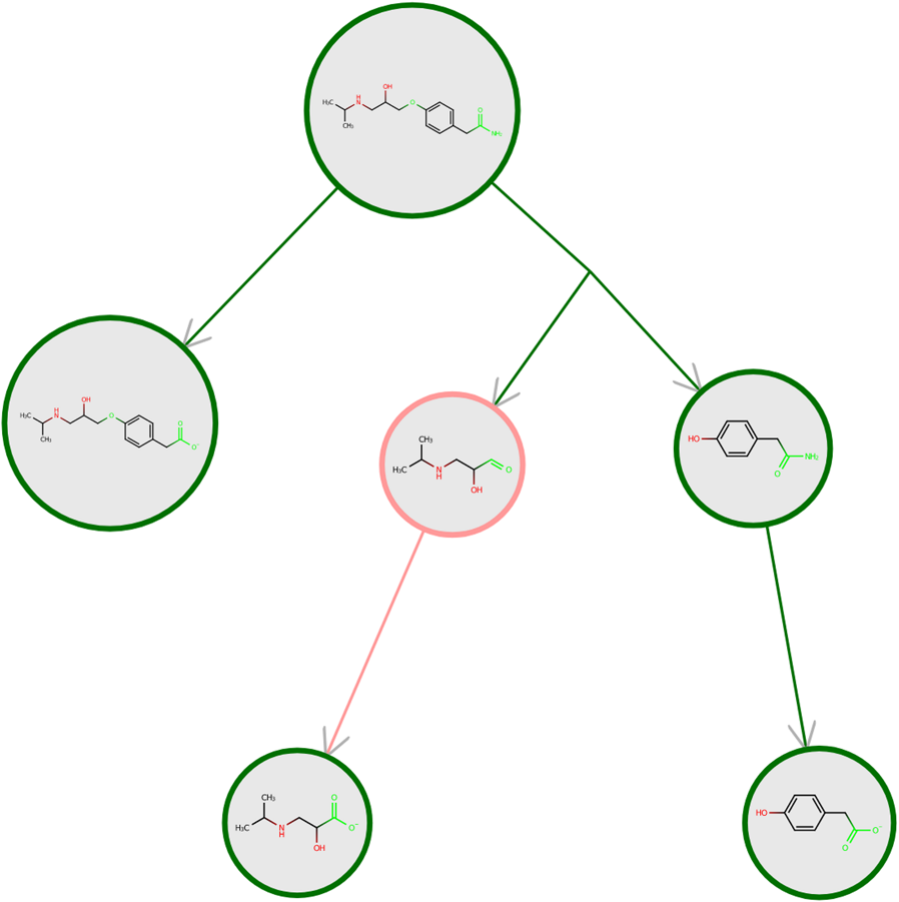
Pathway prediction of Atenolol with our applicability domain implementation. We highlight the applicability domain assessment for compounds, functional groups, and transformations in the pathway view. The reactivity centers in the structure – red marking means no rules trigger this activity center, green marking means the reactivity center is covered by at least one rule. A green circle around the compound gives compounds within the applicability domain, a red circle identifies compounds out of the applicability domain. The colour of the edges show if the reliability or local compatibility is above (green) or below (red) the chosen threshold

Prediction accuracy of the pathway prediction engine drops substantially for molecules that differ greatly from known data. Applicability concepts are important to estimate whether the model interpolates within known training data or extrapolates to unseen chemical space [18, 19]. For the prediction of reactions, not only the molecular structure of the reactant is important, but also the transformation itself [20]. Additionally, for rule-based prediction models the predicted transformations are restricted to the set of transformation rules (templates) and tools have to be developed, indicating when a molecule might be insufficiently covered by the set of rules. In the multilabel setting in enviPath. where multiple transformation rules are predicted, there may be no applicability domain where all transformations can be accurately predicted. To address this, a tailored applicability domain has been developed for enviPath that assesses the reliability of a new compound’s predicted pathway for each individual transformation (visualized in Fig. 6), leading to more accurate predictions and the development of more effective biotransformation models [16].

Our approach [16] provides two applicability domain assessments on the compound level and two assessments on the transformation level. For all compounds we calculate an applicability score that indicates whether the compound is similar to the training set, in terms of fingerprints and compatible rules. Additionally, all functional groups containing hetero atoms are highlighted in green (red), indicating that they are (not) sufficiently represented in the training set. For each transformation, we provide a reliability score and a local goodness of fit. The reliability score represents the average similarity of the *n* most similar compounds in the training set for which the same transformation rule applies, while the local goodness of fit is the ratio of correct predictions for these *n* compounds. The parameter *n* can be set by the model developer.

The evaluation of the applicability domain criteria can be visualized in the pathway view (see Fig. 6) and is documented on the compound pages.

#### enviLink—associating biotransformation rules with EC classes

Understanding contaminant degradation in environmental microbial communities ultimately requires an understanding of which enzymes can catalyze specific biotransformation reactions given a specific chemical structure. Shotgun sequencing of DNA or RNA extracted from microbial communities (i.e., metagenomic and metatranscriptomic data) produces data that contains information on genes or gene transcripts encoding for specific enzymes. This information could ideally be used to predict the biotransformation functions of the microbial community [21]. To this end, tools have been developed that allow predicting potentially catalyzing enzymes for a given biochemical reaction, as defined by substrates and products [11, 13, 22, 23]. However, these tools have been mostly trained on databases focusing on the metabolism of compounds produced by nature (e.g., KEGG). They are therefore of limited utility to predict enzymes involved in the biotransformation of xenobiotic compounds, which contain many functional groups foreign to natural metabolic pathways.

In contrast, the *Eawag-BBD* package in enviPath exclusively contains information on experimentally observed contaminant biotransformation reactions, which have also served as a basis for deriving the generalized biotransformation rules used in enviPath for pathway prediction [5]. Most contaminant biotransformation reactions in *Eawag-BBD* are annotated with an EC number, which has been manually extracted by a data curator from the original publication reporting the experimental evidence. Most reactions are annotated with a 4th or 3rd level EC number (44.2% and 43.3%, respectively).

**Fig. 7:**
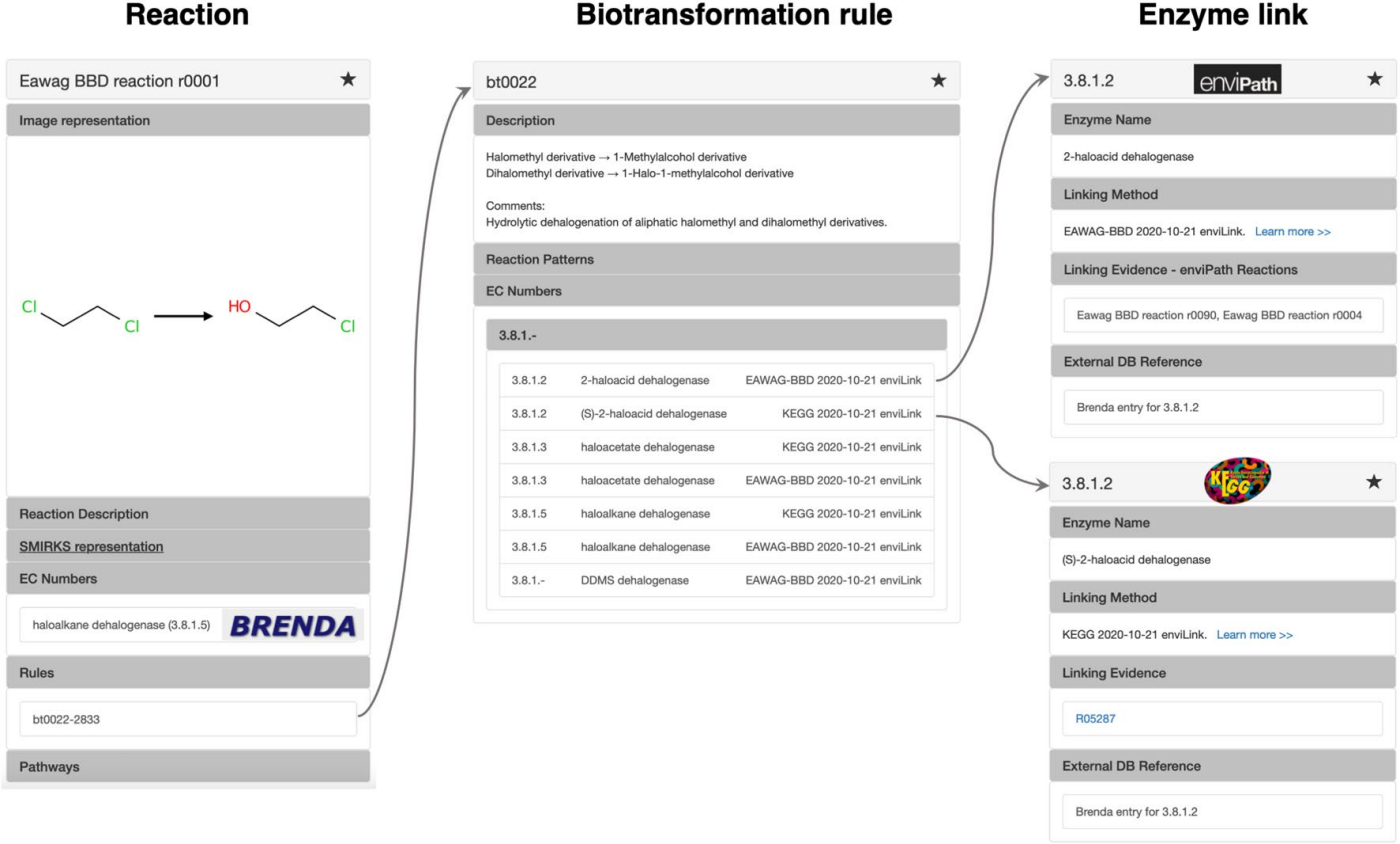
enviLink connects reactions with biotransformation rules. Each rule is associated with a list of enzymes that can catalyze the generalized biotransformation reaction encoded by the rule. Listed enzymes are linked to internal enviPath reactions or external KEGG reactions

We used the *Eawag-BBD* data and KEGG to develop enviLink, a new resource providing linkages between generalized biotransformation rules and 3rd-level EC classes. We developed the rule-EC linkages provided in enviLink in three steps (see Fig. 7) [14]: (i) Application of all *Eawag-BBD* biotransformation rules on *Eawag-BBD* and KEGG compounds; (ii) Comparison of "in silico" generated reaction pairs (i.e., substrate(s) and product(s)) with *Eawag-BBD* or KEGG reactions to find matching reactions; and (iii) establishing rule-enzyme links by associating the enzyme class of a matching reaction with the rule that predicted this reaction. Finally, to derive linkages between generalized rules and 3rd level EC classes, 4th level EC numbers were summarized into the corresponding 3rd-level EC classes. This analysis resulted in 316 derived linkages between rules used for contaminant biotransformation prediction in enviPath and 3rd level EC classes. 32.6% of the identified rule-EC linkages overlap between the two databases, whereas 40.2% and 27.2%, respectively, are originating from *Eawag-BBD* and KEGG only. The fact that more than one third of the linkages originate from *Eawag-BBD* exclusively demonstrates its unique information content with respect to contaminant biotransformation. For selecting top enzymes from the predicted enzyme candidates and comparison with other enzyme prediction tools, we will implement a ranking metric similar to related work in the future [11, 13, 22, 23]. enviLink is encoded in RDF triples as part of the enviPath RDF database. enviLink is available online.^3^

#### Runtime improvements

To enhance the runtime performance of the enviPath server, we have implemented several optimizations. Firstly, we have increased the maximum heap space for the Tomcat server from 2 GB to 16 GB. Secondly, we have optimized the data initialization and data query of rules in enviPath to enable faster pathway predictions. Specifically, we have replaced the dynamic querying of applicable rules from data packages with a lazy initialization approach that searches applicable rules only once and stores the results for subsequent downstream predictions, each downstream prediction then only needs to load the rules that are not queried yet and needed for this step. The specific runtime improvements gained by these improvements are hard to quantify due to the incremental and complex nature of the implementation. However, as example we can consider the loading of multiple objects from the database. This process changed from using individual database queries to one query to get the full set of requested objects in multiple cases. In turn, processing moved from database queries to in-memory operations, which drastically increased the runtime when handling large collections of objects, e.g. when applying transformation rules in the prediction step.

### Implementation

Besides a new and improved prediction engine, we improved the implementation of the system, adding new features to both access and manipulate data. We created a plugin system that eases the addition of new features for specific use cases in the future, we implemented a client library that allows users to integrate enviPath data and predictions directly into their code, and implemented a feature that enables users to merge packages, simplifying data integration workflows.

#### Plugin infrastructure

We opened the enviPath implementation and included more functionality via wrappers and plugins. As a first extension, we included the functionality of RDKit [24]. For example, we can now directly use reaction SMARTS via RDKit, which was unavailable due to limitations in the Ambit SMIRKS library [25]. In general, the plugins are available as tools to calculate descriptors or as classifiers in the prediction engine, making a large number of RDKit descriptors available for the classification process as well as offering new methods for the prediction engine. Besides RDKit, we implemented a plugin that includes Biotransformer [26] as an option for the prediction engine.

#### Client library

**Fig. 8:**
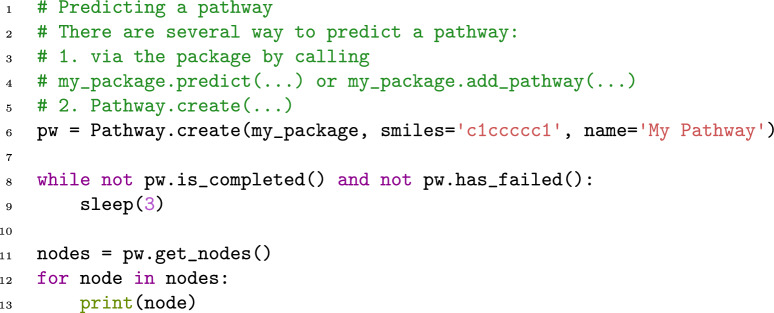
Example use of the python library to predict the pathway of a compound. The detailed code with all prerequisites can be found in our GitHub repository

To ease the use of enviPath in existing pipelines, we implemented a library in Python that offers the functionality of enviPath in a convenient interface [27]. The library is available at https://github.com/enviPath/enviPath-python. Figure 8 gives a short example code how a compound is submitted to the prediction engine and the resulting pathway is retrieved. The library is able to both access the data as well create new data and access the prediction engine.

#### Merge packages

Finally, we added a feature to merge several packages into one. This allows the user to have a working main package and at the same time add new data without compromising the quality of the main package until the import is complete. Once a new package is finalized and all data is added, the new package can be merged into the old one.

### Outlook

Currently, a tool for automatic extraction of rules from biotransformation reactions is in development (enviRule) [28]. enviRule can automatically cluster reactions into different groups based on reaction fingerprints, and extract rules from them. The genericity of rules is optimized against the downstream transformation product prediction task, thus guaranteeing a good prediction performance when used for training predictive models. enviRule also offers functionalities to update the automatic rules once new reaction data have been added to enviRule.

We further are working on implementing methods to identify and mitigate bias in chemical databases into enviPath [29]. In this process we will implement a visualization that can highlight the relationships among the compounds and potential biases and ways to mitigate them. Identifying gaps and mitigating them by adding new data will help to grow the applicability domain of the models and extend the usability into new domains.

## Conclusions

Over the last 8 years, enviPath has become the standard resource for environmental contaminant biotransformation pathways. Besides a large number of improvements in usability and speed, we extended both scope of the database and functionality of the prediction engine. We added two new data sets, *Eawag-Soil* and *Eawag-Sludge*, as well as links to enzymatic processes via enviLink. In terms of prediction engine, we improved the evaluation by considering a more holistic view of the predicted pathways, and implemented an applicability domain function specifically for the prediction of metabolic pathways. In the future, we will further improve the prediction engine and plan to include further data sets to extend the scope of both the data and trained models.

## Data Availability

All data presented in this paper are published at https://envipath.org under a Creative Commons Attribution-NonCommercial-ShareAlike 4.0 International license.

## Abbreviations

Eawag-BBD: Eawag Biocatalysis/Biodegradation Database
Eawag-Soil: Eawag Soil dataset [9]
Eawag-Sludge: Eawag Sludge dataset [10]
enviLink: Database linking contaminant biotransformation rules to enzyme classes [14]
enviRule: Tool for automatic extraction of rules from biotransformation reactions [28]
KEGG: Kyoto encyclopedia of genes and genomes [2, 4]
RDF: Resource description framework
EC number: Enzyme commission number
SMARTS: SMILES arbitrary target specification
SMILES: Simplified molecular-input line-entry system

## Footnotes

1 The enviLink data can be found at online at enviPath: https://envipath.org/package/32de3cf4-e3e6-4168-956e-32fa5ddb0ce1/rule.

2 seehttps://wiki.envipath.com/doku.php?id=changelog_0.3.0.

3 see https://github.com/emanuel-schmid/enviLink.
